# Bioguided Isolation of (*E*)-Ethyl-12-cyclohexyl-4,5-dihydroxydodec-2-enoate from the Aerial Parts of *Heliotropium indicum* and Evaluation of Its Mechanism of Action Using the Formalin Test

**DOI:** 10.3390/pharmaceutics18060714

**Published:** 2026-06-10

**Authors:** María Elena Sánchez-Mendoza, Jesús Arrieta, Yaraset López-Lorenzo, Gisela Gutiérrez-Iglesias, Osmar Antonio Jaramillo-Morales, Josué Vidal Espinosa-Juárez

**Affiliations:** 1 Escuela Superior de Medicina, Instituto Politécnico Nacional, Plan de San Luis y Díaz Mirón, Colonia Casco de Santo Tomás, Miguel Hidalgo, México City 11340, Mexico; 2 Departamento de Enfermería y Obstetricia, División de Ciencias de la Vida, Campus Irapuato-Salamanca, Universidad de Guanajuato, Irapuato 36500, Guanajuato, Mexico; 3 Escuela de Ciencias Químicas, Universidad Autónoma de Chiapas, Ocozocoautla de Espinosa 29140, Chiapas, Mexico

**Keywords:** pain, antinociception, natural product

## Abstract

**Background/Objectives**: *Heliotropium indicum (H. indicum)* is a medicinal plant traditionally used for conditions associated with inflammation, but its active antinociceptive constituents remain poorly defined. This study evaluated the antinociceptive activity of the aerial parts of *H. indicum* through a bioassay-guided approach and explored the mechanism of action of the active compound isolated in the formalin test. **Methods**: Three extracts of *H. indicum* (hexane, dichloromethane, and methanol) were evaluated in male Swiss albino CD-1 mice using the formalin test. The most active extract was fractionated, and its major fractions were screened for antinociceptive activity. Based on the active fraction and previous phytochemical data, (*E*)-ethyl-12-cyclohexyl-4,5-dihydroxydodec-2-enoate (ECDE) was selected for further pharmacological evaluation in the same model. Antagonist pretreatments were used to investigate the involvement of opioid, serotonergic, gamma-aminobutyric acid (GABA_A_), and Nitric Oxide (NO)–soluble Guanylyl Cyclase (sGC) pathways. **Results**: The three extracts reduced nociceptive behavior, mainly during phase II of the formalin test, whereas the dichloromethane extract showed the broadest activity profile and was selected for fractionation. The six fractions significantly reduced phase II nociception, and fraction F5 was selected for purification. ECDE produced a clear dose-dependent antinociceptive effect in phase II, with minimal effect on phase I, and its efficacy was compared with that of ketorolac, a standard antinociceptive drug. Dose–response analysis estimated a DE_50_ of 0.76 mg/kg for ECDE. Pretreatment with N-nitro-L-arginine methyl ester (L-NAME) and [1,2,4]oxadiazolo [4,3-a]quinoxalin-1-one (ODQ) significantly attenuated the effect of ECDE, whereas naloxone, methiothepin, and bicuculline did not. **Conclusions**: ECDE was identified in *H. indicum* as one of the compounds contributing to this effect. Its activity appears to be directed mainly toward inflammatory nociception and to depend, at least in part, on the NO–sGC pathway.

## 1. Introduction

Pain remains one of the most prevalent and disabling health problems worldwide, and inflammatory pain in particular continues to impose a substantial burden on both individuals and public health. Recent population-level data indicate that chronic pain affects a large proportion of adults, underscoring the persistent need for safer analgesics with greater mechanistic diversity [1]. Current pharmacological management of pain relies mainly on nonsteroidal anti-inflammatory drugs (NSAIDs), acetaminophen, opioids, and adjuvant agents selected according to the underlying mechanism and clinical context. However, these options remain suboptimal for many patients. NSAIDs are widely used and effective in inflammatory pain states, but their use is limited by gastrointestinal, renal, and cardiovascular toxicity, particularly with prolonged exposure or in susceptible populations [2]. Opioids, while indispensable in selected scenarios, are also constrained by tolerance, dependence, misuse liability, and other adverse effects [3]. Together, these limitations have intensified interest in the identification of new analgesic agents with an improved efficacy–safety profile and, ideally, novel or multimodal mechanisms of action.

In this context, medicinal plants continue to represent a valuable source of pharmacologically active compounds and structurally diverse scaffolds for analgesic drug discovery [4]. Their relevance in pain research lies not only in their chemical diversity but also in their potential to modulate multiple biological targets involved in nociception, including inflammatory mediators, ion channels, and neurotransmission pathways. Moreover, plants used in traditional medicine provide a rational basis for pharmacological investigation, particularly when ethnomedical knowledge is complemented by experimental validation and the isolation of active constituents. Accordingly, botanicals traditionally employed for pain and inflammation-related conditions remain an important avenue for the identification of novel analgesic agents [5].

*Heliotropium indicum* (Boraginaceae) is a widely distributed medicinal herb used in traditional medicine across Africa, Asia, and Latin America. Ethnopharmacological surveys have documented its use in conditions associated with pain and inflammation [6,7,8]. Experimental evidence has further shown that extracts of *H. indicum* possess anti-inflammatory activity, reducing inflammatory-cell infiltration, protein extravasation, and the levels of Tumor Necrosis Factor-alpha (TNF-α), Prostaglandin E2, and Monocyte chemoattractant protein-1 in lipopolysaccharide-induced uveitis [9]. These findings suggest the hypothesis that this plant contains constituents capable of modulating pathways closely linked to nociceptive processing, especially those associated with inflammatory pain.

Based on this rationale, the present study investigated the antinociceptive potential of *H. indicum* through a bioactivity-guided approach. The research progressed from crude extracts to fractions and subsequently to the isolation of an active molecule. In addition, its mechanism of action was pharmacologically explored using the formalin test. This strategy was intended to identify the constituent associated with the observed activity and to contribute to the pharmacological validation of this species as a potential source of antinociceptive agents.

## 2. Materials and Methods

### 2.1. Animals

Male Swiss albino CD-1 mice (25–30 g) were used. Animals were obtained from the School of Chemical Sciences, Autonomous University of Chiapas, and housed under standard laboratory conditions (24 ± 2 °C) with a 12 h light/12 h dark cycle. Food and water were available ad libitum prior to treatment. Each mouse was used in a single experimental session and was euthanized immediately by an overdose of sodium pentobarbital (200 mg/kg, i.p.), and death was confirmed by the absence of respiration and cardiac activity in accordance with the Official Mexican Standard NOM-062-ZOO-1999 (Technical Specifications for the Production, Care, and Use of Laboratory Animals) [10]. All procedures were approved by the Research Committee of the Universidad Autónoma de Chiapas (approval date: 4 February 2025; protocol number: 03/ECQ/RPI/003/25).

### 2.2. Drugs and Reagents

The solvents (hexane, dichloromethane, methanol and ethyl acetate), analytical/HPLC grade, and formaldehyde were obtained from J.T. Baker (Radnor, PA, USA). Naloxone, methiothepin, glibenclamide, ODQ, L-NAME, and bicuculline were purchased from Sigma-Aldrich (St. Louis, MO, USA). Ketorolac (AMSA Laboratories, Mexico City, Mexico) was used as the reference drug.

### 2.3. Plant Material

*Heliotropium indicum* was collected in the municipality of Copainalá, Chiapas State, Mexico, in July 2024. A specimen from the original collection is located in the Chip Herbarium of the Dr. Faustino Miranda Garden Botanical in Tuxtla Gutiérrez, Chiapas, with registration number 27,855. The taxonomic classification was conducted by Manuel de Jesús Gutiérrez Morales, biologist, at Botanical Garden of the Chiapas State.

### 2.4. Experimental Design

The study was conducted using a sequential bioassay-guided approach, in which antinociceptive activity was tracked from crude extracts through the isolation and evaluation of an active compound. The work was organized into four stages: the first consisted of screening three extracts (hexane, dichloromethane, and methanol); the second involved the evaluation of six major fractions obtained from the most active extract; the third comprised the pharmacological assessment of the isolated compound; and the final stage explored its mechanism of action through pretreatment with pharmacological antagonists.

Animals were allocated to groups using a random sequence generated before the experiment, and treatments were coded by a different investigator so that behavioral scoring was performed blind to treatment. The order in which animals were evaluated was also randomized to avoid potential effects related to time of day or testing sequence. In addition to the vehicle group, a reference drug group was included.

### 2.5. Obtaining Heliotropium indicum Extracts

The aerial parts of *H. indicum* (leaves and stems) were extracted by successive maceration, as previously described by López-Lorenzo et al. [11]. The solvents were selected according to increasing polarity in order to obtain extracts with different phytochemical profiles. Hexane was used to extract mainly non-polar constituents, dichloromethane was used as an intermediate-polarity solvent to recover moderately polar constituents, whereas methanol was selected to extract more polar compounds. This sequential extraction strategy was intended to provide a broad preliminary separation of the constituents of *H. indicum* and to allow for comparison of their antinociceptive activity according to solvent polarity. A glass container was filled with 7.6 kg of previously dried and ground plant material, and 45 L of hexane was added. The mixture was left to stand for three days at room temperature (22 ± 2 °C), after which it was filtered and concentrated under reduced pressure using a rotary evaporator. This procedure was repeated twice. The plant residue was then extracted successively with dichloromethane and methanol using the same methodology. This process yielded hexane (202 g), dichloromethane (227 g), and methanol (200 g) extracts.

### 2.6. Fractionation of the Most Active Extract and Isolation of the Active Compound

The dichloromethane extract exhibited the highest biological activity, as determined by the assessments of the extracts in the formalin model. As a result, 220 g of this extract was subjected to silica gel column chromatography using a gradient with broad polarity changes to obtain six fractions (F1–F6). The elution system consisted of 100% hexane, a hexane/ethyl acetate mixture in varying proportions (9:1, 8:2, 7:3, and 5:5), and 100% ethyl acetate. The fraction with the highest biological activity was F5. The F5 fraction was subsequently separated by using the elution system of 100% hexane, a hexane/ethyl acetate mixture in proportions (95:5, 90:10, 85:15, 80:20, 75:25 and 70:30). 30 mL fractions were collected. Subfractions 80–110, eluted with hexane/ethyl acetate (9:1), were combined because they showed the same Rf value by thin-layer chromatography, suggesting the presence of the same major constituent. The pooled subfractions were further purified by crystallization using a hexane/ethyl acetate mixture (8:2), yielding a white solid with a melting point of 87–88 °C. The compound was identified by comparison of its ^1^H- and ^13^C-NMR data with those previously reported, as well as by electrospray ionization (ESI) mass spectrometry [11]. The final compound demonstrated antinociceptive activity when tested in the formalin model.

### 2.7. Treatment Preparation and Administration

The extracts, fractions, and isolated compound were suspended in 1% Tween 80 in distilled water and administered orally at a volume of 1 mL/100 g of body weight. All treatments were given 30 min before formalin injection. The hexane and dichloromethane extracts were tested at doses of 3.16, 10, 31.6, and 100 mg/kg, whereas the methanol extract was administered at 0.1, 1.0, 10, and 100 mg/kg. The fractions obtained from the dichloromethane extract were evaluated at a single dose of 100 mg/kg. The isolated compound was tested at 0.01, 0.1, 1.0, 10, and 100 mg/kg. Ketorolac was used as the reference drug and was evaluated at doses of 0.1, 0.31, 1.0, and 3.16 mg/kg.

### 2.8. Formalin Test

The formalin test was performed according to a standard protocol [12]. Briefly, formalin (2%, 20 µL) was injected subcutaneously into the dorsal surface of the right hind paw using a 1 mL syringe fitted with a 30 G × 13 mm needle. Immediately after injection, each animal was placed in a transparent observation chamber. The number of flinches of the injected paw was recorded as the nociceptive response.

Two periods were analyzed: phase I (0–10 min) and phase II (15–60 min), classically interpreted as an early nociceptive phase and a later phase with a greater inflammatory component. Behavioral assessments were conducted for 1 min every 5 min. The antinociceptive effect was expressed as the area under the curve (AUC), calculated using the trapezoidal method [13]. The percentage of antinociception was calculated using the following formula:

$$\%\;Antinociception=\frac{AUC\;\left(Control\;group\right)-AUC\;\left(Experimental\;group\right)}{AUC\;\left(Control\;group\right)}\times 100$$

### 2.9. Pharmacological Antagonism Study

To investigate the mechanisms potentially underlying the antinociceptive effect of (*E*)-ethyl-12-cyclohexyl-4,5-dihydroxydodec-2-enoate, several pain-modulating pathways were explored using pharmacological antagonists. Mice received vehicle or the corresponding pretreatment drug 15 min before administration of the isolated compound (1 mg/kg, p.o.) The pretreatments included naloxone (5 mg/kg, i.p.; non-selective opioid receptor antagonist), methiothepin (1 mg/kg, i.p.; non-selective serotonin receptor antagonist), L-NAME (10 mg/kg, i.p.; non-selective nitric oxide synthase inhibitor), ODQ (0.1 mg/kg, i.p.; soluble guanylate cyclase inhibitor), and bicuculline (1.0 mg/kg, i.p.; GABA_A_ receptor antagonist). Dose selection and timing were based on previously published protocols [14,15,16,17,18]. Following pretreatment and administration of the isolated compound, antinociceptive responses were measured and compared with those observed in animals receiving the isolated compound alone.

### 2.10. Statistical Analysis

Results are presented as mean ± SEM (n = 8 per group). Nociceptive behavior was summarized as time-course profiles (time vs. number of flinches) and the AUC. The AUC was used as an integrated measure of the nociceptive response over time; reductions in AUC relative to the vehicle group were interpreted as antinociceptive effects. Data normality was assessed using the Shapiro–Wilk test. When the assumption of normality was met, multiple-group comparisons were performed using one-way ANOVA. Comparisons of treatment groups versus the vehicle group were analyzed using Dunnett’s post hoc test, whereas pairwise comparisons among all groups were performed using Tukey’s multiple-comparisons test when required. For datasets that did not meet normality criteria, the Kruskal–Wallis test was applied, followed by Dunn’s post hoc test. Statistical analyses were performed using GraphPad Prism (version 8.0; GraphPad Software, San Diego, CA, USA). A *p* value < 0.05 was considered statistically significant.

## 3. Results

### 3.1. Antinociceptive Evaluation of Heliotropium indicum Extracts

**Figure 1:**
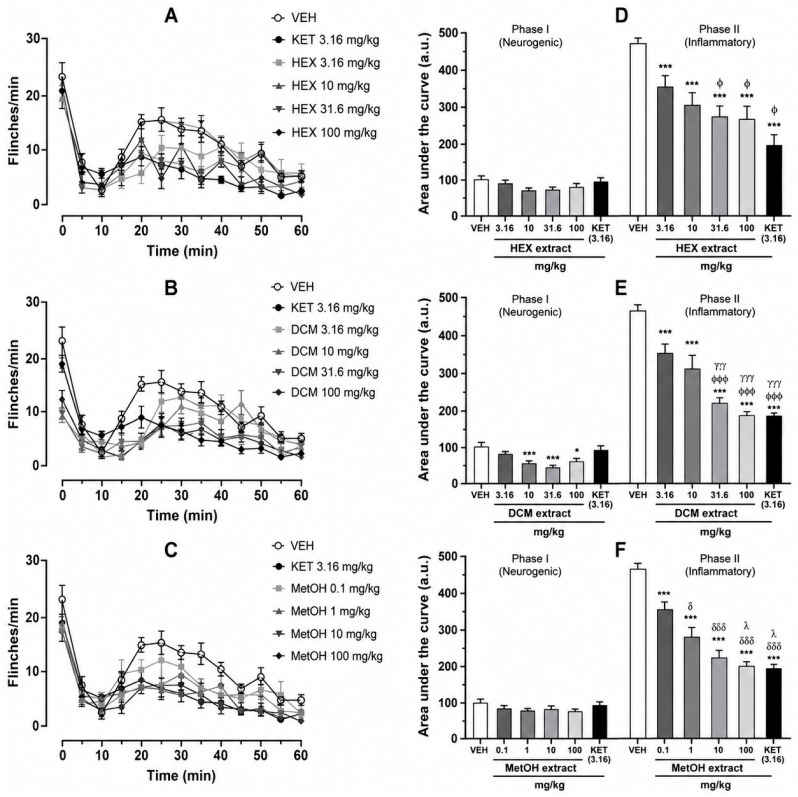
Antinociceptive effect of *Heliotropium indicum* extracts in the formalin test. For each extract, antinociceptive activity was evaluated by time-course analysis of nociceptive behavior and by area under the curve (AUC) quantification in Phase I (0–10 min) and Phase II (15–60 min). Panels (**A**,**D**), (**B**,**E**), and (**C**,**F**) correspond to the HEX, DCM, and MetOH extracts, respectively. Ketorolac (KET, 3.16 mg/kg, p.o.) was used as the reference drug. Data are expressed as mean ± SEM (n = 8). Time-course data were analyzed by two-way ANOVA followed by Dunnett’s post hoc test; AUC data were analyzed by one-way ANOVA followed by Tukey’s post hoc test. * *p* < 0.05 and *** *p* < 0.001 indicate significant differences compared to the vehicle group; ^ϕ^ *p* < 0.05 and ^ϕϕϕ^ *p* < 0.001 vs. 3.16 mg/kg; ^γγγ^ *p* < 0.001 vs. 10 mg/kg for HEX and DCM extract; ^δ^ *p* < 0.05 and ^δδδ^ *p* < 0.001 vs. 0.1 mg/kg, ^λ^ *p* < 0.05 vs. 1 mg/kg for the MetOH group treatment.

Intraplantar formalin administration in the vehicle (VEH) group produced the characteristic biphasic nociceptive response, with an initial peak in flinching behavior immediately after injection (0 min), followed by a quiescent period and a second phase of sustained activity between 15 and 60 min (Figure 1A–C).

The hexane (HEX) extract produced a modest antinociceptive effect in the formalin test, with significant reductions in nociceptive behavior at 31.6 mg/kg during minutes 25–35 and at 100 mg/kg at minutes 25 and 35 (*p* < 0.01 vs. vehicle) (Figure 1A). This limited effect during the time-course analysis was consistent with the AUC results, as the HEX extract did not significantly modify Phase I (0–10 min). By contrast, in Phase II (15–60 min), the VEH group exhibited an AUC of 464.7 ± 42.18 arbitrary units (a.u.), and the HEX extract significantly reduced this parameter from the lowest dose tested (*p* < 0.001 vs. vehicle) (Figure 1D).

Among the three extracts, the dichloromethane (DCM) extract showed the most pronounced antinociceptive profile. Notably, doses of 10 and 31.6 mg/kg were the only treatments that significantly decreased the early nociceptive response from the first observation point (9.0 ± 1.2 and 9.3 ± 1.1 flinches, respectively; *p* < 0.01 vs. vehicle). During Phase I, the DCM extract produced a sustained inhibition of nociceptive behavior (Figure 1B). In addition, in phase II, the 100 mg/kg markedly decreased the number of flinches to 1.1 ± 0.2 at minute 15 (*p* < 0.01 vs. vehicle), and remained significantly lower than that of the vehicle group during minutes 15–40 (*p* < 0.05). In agreement with the time-course data, the DCM extract was the only treatment that significantly reduced the AUC during Phase I, with significant effects observed from 10 mg/kg onward (*p* < 0.001 vs. vehicle) (Figure 1E). During Phase II, this extract also exerted the strongest effect, significantly reducing the AUC from 3.16 mg/kg onward (*p* < 0.0001 vs. vehicle). Moreover, the 31.6 and 100 mg/kg doses were significantly more effective than 10 mg/kg, supporting a clear dose-dependent effect during the inflammatory phase.

In contrast, the methanol (MetOH) extract did not alter Phase I of the formalin test, but it significantly reduced nociceptive behavior during the inflammatory phase, particularly between minutes 20 and 50 (Figure 1C). Significant decreases were observed from 1.0 mg/kg onward; for instance, at minute 20, the number of flinches was reduced to 7.2 ± 1.3 (*p* < 0.01 vs. vehicle). At 10 and 100 mg/kg, this inhibitory effect was greater and remained significant at several time points during Phase II (*p* < 0.05 at minutes 20–35). Consistent with these findings, the MetOH extract did not significantly modify the AUC during Phase I, whereas in Phase II it produced a marked reduction from 0.1 mg/kg onward (*p* < 0.001 vs. vehicle) (Figure 1F). In addition, the higher doses produced greater inhibition than the lowest dose, further supporting a dose-dependent effect during this phase. Ketorolac (3.16 mg/kg), used as the reference drug, significantly reduced nociceptive behavior from minutes 10 to 50, as expected.

### 3.2. Antinociceptive Effect of Fractions Obtained from the DCM Extract

**Figure 2:**
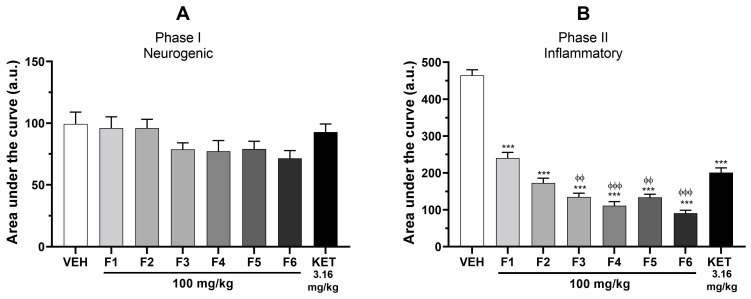
Antinociceptive activity of fractions obtained from the DCM extract in the formalin test. (**A**) Area under the curve (AUC) during phase I (0–10 min). (**B**) AUC during phase II (15–60 min). Fractions were evaluated at 100 mg/kg. Data are presented as mean ± SEM (n = 8). Statistical analysis was performed by one-way ANOVA followed by Tukey’s multiple-comparisons. *** *p* < 0.001, vs. vehicle (VEH); ^ϕϕ^ *p* < 0.01 and ^ϕϕϕ^ *p* < 0.001 vs. ketorolac (KET).

Based on the extract-screening results, the DCM extract, which showed the most prominent antinociceptive activity, was selected for fractionation. The six major fractions obtained from this extract were subsequently evaluated in the formalin test at 100 mg/kg. In phase I, none of the fractions significantly modified the early nociceptive response, as no statistically significant differences were detected relative to the vehicle group (Figure 2A). These findings indicate that fractionation of the parent extract did not yield fractions with detectable activity during the neurogenic phase under the conditions tested.

In contrast, in phase II, the six fractions significantly reduced the nociceptive response relative to the vehicle group. The corresponding AUC values were 240.3 ± 43.7 a.u. for F1, 172.5 ± 37.55 a.u. for F2, 134.7 ± 29.37 a.u. for F3, 111.3 ± 31.23 a.u. for F4, 133.4 ± 24.98 a.u. for F5, and 90.63 ± 23.02 a.u. for F6, whereas ketorolac yielded an AUC of 200.6 ± 36.68 a.u. The fractions differed significantly from the vehicle group (*p* < 0.001 for all comparisons) (Figure 2B), indicating that the antinociceptive activity of the DCM extract was retained after fractionation and was concentrated in the late inflammatory phase.

Post hoc comparisons further revealed differences in efficacy among fractions. F2 produced a significantly greater reduction than F1 (*p* = 0.01), and F3, F4, F5, and F6 were all more effective than F1 (*p* < 0.001 for all comparisons). In addition, no significant differences were detected among F3, F4, F5, and F6. When compared with ketorolac, F1 and F2 did not differ significantly from the reference drug, while F3, F4, F5, and F6 produced significantly lower AUC values than ketorolac (*p* = 0.0072, *p* < 0.0001, *p* = 0.0057, and *p* < 0.0001, respectively).

Overall, these findings indicate that fractionation of the DCM extract preserved and enhanced the antinociceptive activity observed in the inflammatory phase of the formalin test, with the strongest effects observed for fractions F3–F6. F5 was selected for further purification based on its antinociceptive activity together with the fraction yield.

### 3.3. Identification of the Active Compound

**Figure 3:**
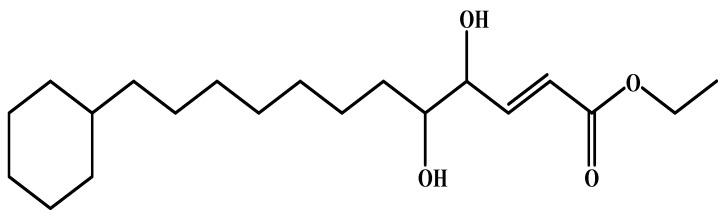
Structure of (*E*)-ethyl-12-cyclohexyl-4,5-dihydroxydodec-2-enoate.

The compound was identified by comparison with previously reported ^1^H- and ^13^C-NMR data and electrospray ionization (ESI) mass spectrometry. ESI (+) showed a molecular ion at *m*/*z* 341.2699 [M + H]^+^ which is consistent with the molecular formula C_20_H_36_O_4_ (*m*/*z*), which corresponds to (*E*)-ethyl-12-cyclohexyl-4,5-dihydroxydodec-2-enoate (Figure 3), which was previously isolated by our work group [11].

### 3.4. Antinociceptive Effect of (E)-Ethyl-12-cyclohexyl-4,5-dihydroxydodec-2-enoate in the Formalin Test

**Figure 4:**
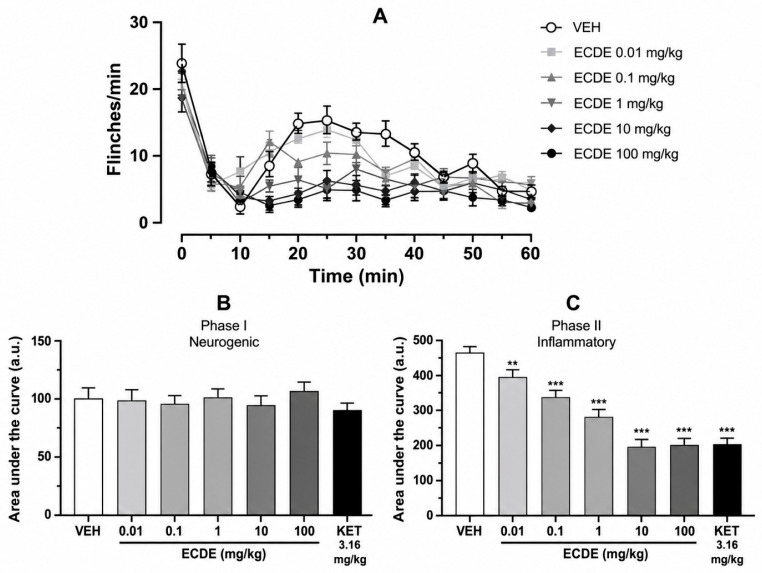
Antinociceptive effect of (*E*)-ethyl-12-cyclohexyl-4,5-dihydroxydodec-2-enoate (ECDE) in the formalin test. (**A**) Time-course of nociceptive behavior following treatment. (**B**) Area under the curve (AUC) during phase I (0–10 min). (**C**) AUC during phase II (15–60 min). Data are expressed as mean ± SEM (n = 8). Statistical analysis was performed using one-way ANOVA followed by Dunnett’s post hoc test for comparisons versus the vehicle group. ** *p* < 0.01 and *** *p* < 0.001 vs. vehicle.

The ECDE was evaluated in the formalin test at doses ranging from 0.01 to 100 mg/kg. In the time-course analysis, ECDE did not produce a clear effect during the early phase. In contrast, its antinociceptive activity became more evident during the inflammatory phase. The dose of 0.1 mg/kg significantly reduced flinching behavior at 20, 25, and 40 min, whereas higher doses produced significant effects over a broader interval, from 25 to 45 min (Figure 4A).

ECDE did not significantly modify phase I AUC values (Figure 4B). In contrast, it produced a marked and dose-dependent reduction in phase II AUC, with values of 395.0 ± 30.79, 334.7 ± 32.79, 276.3 ± 40.85, 190.6 ± 39.47, and 198.4 ± 33.33 a.u. at doses of 0.01, 0.1, 1.0, 10, and 100 mg/kg, respectively. All doses differed significantly from the vehicle group (*p* < 0.01) (Figure 4C). Pairwise comparisons among the lower doses revealed a progressive increase in antinociceptive effect, indicating a clear dose-dependent relationship up to 10 mg/kg. No significant differences were found among 10 mg/kg, 100 mg/kg, and ketorolac, indicating that ECDE reached maximal efficacy at 10 mg/kg and produced an antinociceptive effect comparable to that of the reference drug.

**Figure 5:**
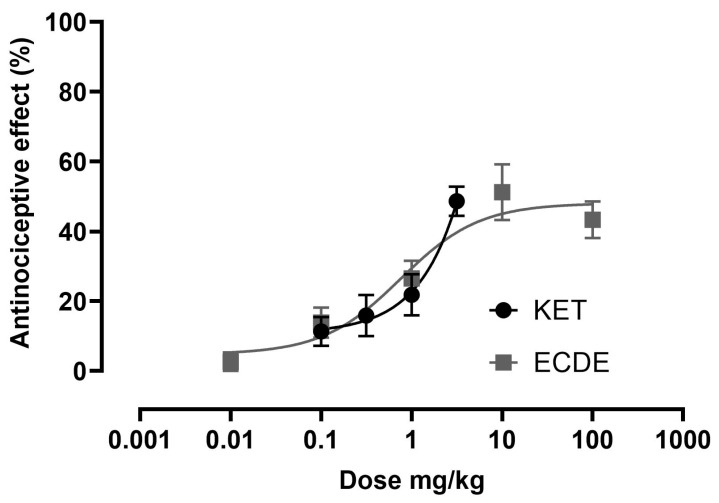
Dose–response curves of ketorolac (KET) and (*E*)-ethyl-12-cyclohexyl-4,5-dihydroxydodec-2-enoate (ECDE) in phase II of the formalin test.

Dose–response analysis revealed a dose-dependent increase in the antinociceptive effect for both ketorolac (KET) and ECDE (Figure 5). Although the ECDE curve showed an apparent leftward shift relative to KET, suggesting a trend toward greater potency, this difference was not statistically supported. The DE_50_ values were 0.76 mg/kg (95% CI: 0.15–2.61) for ECDE and 1.62 mg/kg (95% CI: 0.46–6.27) for KET. Both treatments also showed similar maximal antinociceptive effects. The Emax values were 51.24 ± 7.95% for ECDE and 48.63 ± 4.19% for KET, with no statistically significant differences between them, indicating comparable efficacy. These findings are relevant because they show that ECDE produced antinociceptive effects comparable to those of ketorolac, a well-established reference drug.

### 3.5. Pharmacological Evaluation of the Mechanism of Action of ECDE

To explore the mechanisms underlying the antinociceptive effect of ECDE, mice were pretreated with pharmacological antagonists targeting opioid, serotonergic, GABAergic, and Nitric Oxide (NO)–cyclic Guanosine Monophosphate (cGMP)-related pathways before administration of ECDE. In phase II of the formalin test, the vehicle group showed an AUC of 451.8 ± 47.48 a.u., whereas ECDE alone reduced this value to 260.4 ± 30.85 a.u., confirming its marked antinociceptive effect under these experimental conditions.

**Figure 6:**
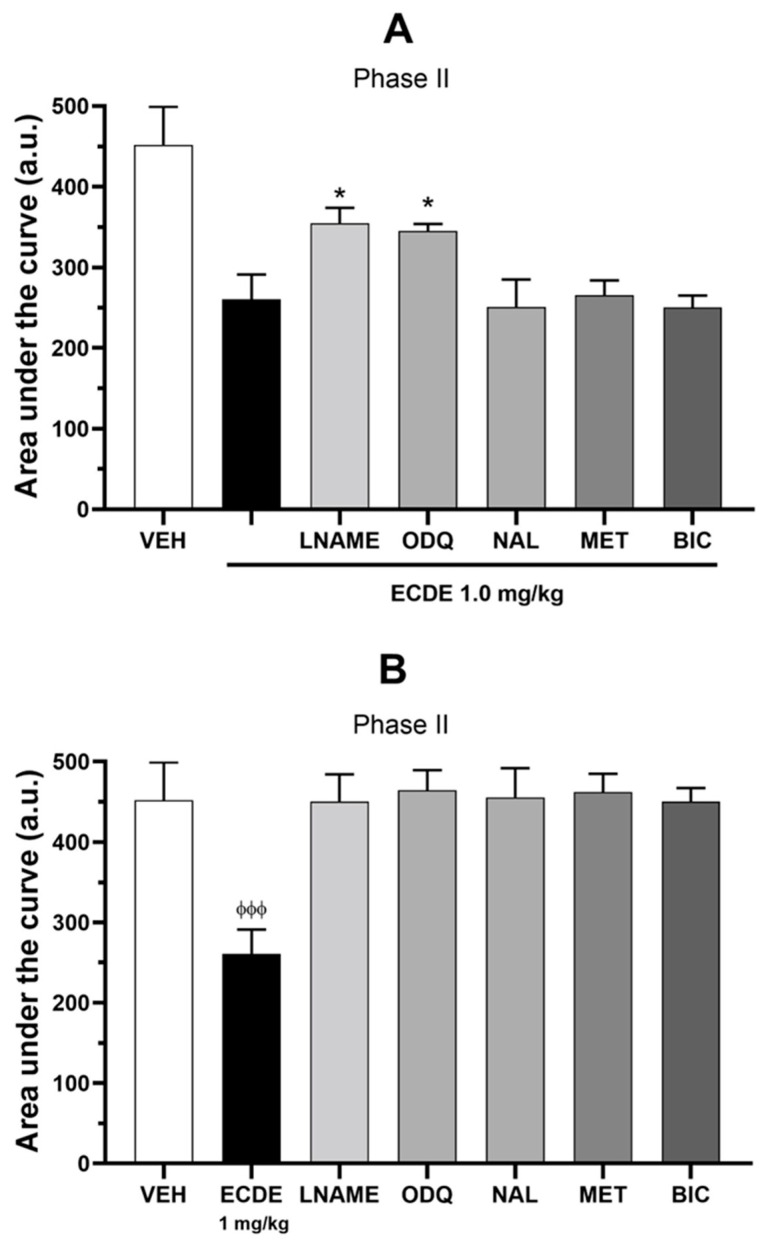
Effect of pharmacological antagonists on the antinociceptive activity of ECDE in phase II of the formalin test. (**A**) Mice were pretreated with L-NAME (10 mg/kg, i.p), ODQ (0.1 mg/kg, i.p), naloxone (NAL, 5 mg/kg, i.p.), methiothepin (MET, 1 mg/kg, i.p.), or bicuculline (BIC, 1 mg/kg, i.p.) before administration of ECDE (1.0 mg/kg, p.o.). (**B**) Effect of the antagonists administered alone on phase II nociceptive behavior. Data are expressed as mean ± SEM. Statistical analysis was performed using one-way ANOVA followed by Dunnett’s post hoc test. * *p* < 0.05 vs. ECDE-treated group in panel (**A**); ^ϕϕϕ^ *p* < 0.001 vs. vehicle in panel (**B**).

Pretreatment with L-NAME or ODQ significantly attenuated the effect of ECDE, increasing the phase II AUC to 354.6 ± 19.02 a.u. and 345.3 ± 8.48 a.u., respectively (*p* < 0.05 vs. ECDE alone). These findings indicate that inhibition of nitric oxide synthase or soluble guanylate cyclase partially reversed the antinociceptive effect of ECDE (Figure 6A).

By contrast, pretreatment with naloxone, methiothepin, or bicuculline did not significantly modify the antinociceptive effect of ECDE. The corresponding AUC values were 250.6 ± 34.42 a.u., 265.3 ± 18.54 a.u., and 250.3 ± 14.54 a.u., respectively, and none differed significantly from the ECDE-treated group. In addition, administration of the antagonists alone did not significantly alter the nociceptive response, indicating that their effects were not attributable to intrinsic actions on formalin-induced behavior (Figure 6B).

## 4. Discussion

The present study shows that *H. indicum* possesses clear antinociceptive activity in the formalin test and that this activity can be traced from the crude extracts to a bioactive fraction and, ultimately, to the isolated compound ECDE. All three extracts exhibited antinociceptive activity, mainly during phase II, indicating that *H. indicum* contains active constituents distributed across extracts of different polarity. Among them, the dichloromethane extract displayed the broadest activity profile, as it not only reduced phase II nociception but also produced detectable effects during the early phase. Interestingly, this phase I activity was not retained after fractionation, since the resulting fractions predominantly inhibited phase II. This finding suggests that the early antinociceptive effect may have depended on the combined action of multiple constituents present in the parent extract [19]. Fractionation may have disrupted additive or synergistic interactions among these compounds, or redistributed those involved in the neurogenic component of nociception, thereby restricting the detectable activity to the inflammatory phase. Thus, the persistence of activity in phase II after fractionation suggests that the compounds enriched in these fractions are more closely related to anti-inflammatory than to early neurogenic mechanisms. The activity observed in fractions F3, F4, and F6 suggests that additional bioactive constituents may also contribute to the antinociceptive effects of *H. indicum*, and these fractions should be prioritized in future phytochemical studies. These findings are also in line with previous evidence showing that *H. indicum* exerts anti-inflammatory activity in vivo [9].

The formalin test remains particularly informative because its biphasic pattern allows for a preliminary distinction between effects on the early nociceptor-driven component and the later phase, which is more strongly influenced by inflammation and central sensitization. Phase I has classically been linked to direct activation of primary afferents, whereas phase II reflects a more complex response involving inflammatory mediators and sensitization of spinal circuits. In the present study, the isolated compound ECDE did not significantly modify phase I AUC values, but it produced a marked and dose-dependent reduction in phase II. Taken together, these findings indicate that ECDE acts predominantly on mechanisms associated with persistent inflammatory nociception rather than on the acute neurogenic component of the formalin response [12].

From a pharmacological perspective, ECDE preserved the principal activity profile observed in the parent dichloromethane extract and its active fractions, characterized by a preferential inhibition of phase II nociception. This consistency strengthens the interpretation that the activity of the dichloromethane extract was not a nonspecific property of the crude mixture but was at least partly attributable to a discrete constituent that remained active after purification. Dose–response analysis further supported this interpretation, showing that ECDE produced a well-defined antinociceptive effect across the tested dose range, the efficacy was compared with the standard antinociceptive drug ketorolac. Notably, ECDE presents a significant pharmacological advantage over conventional NSAIDs like ketorolac: while the latter is limited by its well-documented gastrotoxicity, ECDE combines its potent antinociceptive effect with previously reported oral gastroprotective activity [11]. This dual pharmacological profile underscores the relevance of ECDE as a promising bioactive constituent of *H. indicum* with a superior safety margin for potential therapeutic applications.

The pharmacological antagonism experiments provide a more specific mechanistic framework for the antinociceptive effect of ECDE. Pretreatment with L-NAME and ODQ significantly attenuated its effect, whereas naloxone, methiothepin, and bicuculline did not. Because L-NAME inhibits nitric oxide synthase and ODQ blocks soluble guanylyl cyclase, these findings support the involvement of the NO– sGC– cGMP pathway in the antinociceptive action of ECDE. In pharmacological terms, this suggests that ECDE depends, at least in part, on endogenous nitric oxide production and subsequent activation of soluble guanylyl cyclase, leading to cGMP formation and downstream signaling events that reduce nociceptive transmission [14,20]. This interpretation is consistent with previous evidence showing that the L-arginine/NO/sGC/cGMP cascade contributes to peripheral antinociception in the formalin test and other inflammatory pain models [21,22]. More specifically, nitric oxide generated from L-arginine activates soluble guanylyl cyclase, increasing intracellular cGMP levels, which in turn can engage downstream effectors such as protein kinase G and ion conductance-modulating mechanisms that ultimately reduce neuronal excitability [23]. Thus, the present results place ECDE among the compounds whose antinociceptive effect during inflammatory nociception appears to require an intact NO–sGC–cGMP signaling pathway.

Interestingly, the same compound has previously been shown to exert gastroprotective activity through a mechanism involving nitric oxide, further supporting the pharmacological relevance of nitrergic signaling in the biological actions of ECDE [11].

Some limitations of the present study should also be acknowledged. Although the data strongly support activity in phase II of the formalin test, they do not identify the downstream inflammatory mediators regulated by ECDE. Given the prior evidence for anti-inflammatory effects of *H. indicum*, future studies should examine whether ECDE modulates mediators such as prostaglandins, TNF-α, interleukin-1β, interleukin-6, or other components of the inflammatory cascade [6,7]. In addition, the antinociceptive activity of ECDE was evaluated only using the formalin test. This model was selected because it provides a biphasic nociceptive response, allowing for the assessment of an early neurogenic phase and a later phase with a strong inflammatory component. However, the use of a single pain model limits the extrapolation of the findings to other types of nociception. Therefore, future studies should evaluate ECDE in additional models, such as the hot plate or tail-flick tests for centrally mediated nociception, the acetic acid-induced writhing test for visceral inflammatory pain, and carrageenan-induced inflammatory pain and neuropathic pain models. Finally, additional pharmacokinetic and safety studies will be necessary to determine whether the observed potency in the formalin test translates into a viable pharmacological profile.

## 5. Conclusions

*H. indicum* has been traditionally used for conditions associated with pain and inflammation; however, the constituents involved in its antinociceptive effects remain insufficiently characterized. In this study, the aerial parts of *H. indicum* showed antinociceptive activity in the formalin test, and ECDE produced a dose-dependent effect mainly during the inflammatory phase. The attenuation of this effect by L-NAME and ODQ suggests that the NO–sGC pathway participates, at least in part, in its mechanism of action. Overall, these findings support ECDE as a bioactive constituent with antinociceptive potential and contribute to the pharmacological validation of *H. indicum*. Nevertheless, further studies using complementary pain models, inflammatory mediator analysis, and pharmacokinetic and safety evaluations are needed to better define its therapeutic potential.

## Data Availability

The original contributions presented in this study are included in the article. Further inquiries can be directed to the corresponding authors.
